# A248 THE ROLE OF DIETARY TRYPTOPHAN IN INDOLE AND KYNURENINE PRODUCTION AND IMMUNE MODULATION IN HEALTHY INDIVIDUALS

**DOI:** 10.1093/jcag/gwab049.247

**Published:** 2022-02-21

**Authors:** G H Rueda, N Causada-Calo, A Nardelli, M I Pinto-Sanchez, R Borojevic, J Libertucci, L Loonen, J Wells, H Sokol, E Verdu, P Bercik

**Affiliations:** 1 Medicine, McMaster University, Hamilton, ON, Canada; 3 Biochemistry and Biomedical Sciences, McMaster University, Hamilton, ON, Canada; 4 McMaster University, Hamilton, ON, Canada; 6 Wageningen University & Research, Wageningen, Gelderland, Netherlands; 7 Sorbonne Universite, Paris, Île-de-France, France

## Abstract

**Background:**

Natural supplements are widely consumed by the general public, with little evidence of mechanistic support. Tryptophan has gained central attention, being transformed by host and gut microbial enzymes into multiple bioactive metabolites that regulate immunity and mood. Indoles are activators of the aryl hydrocarbon receptor (AhR), crucial for the maintenance of intestinal homeostasis. Tryptophan has been advocated to prevent chronic inflammatory conditions, however the clinical data to support this are missing.

**Aims:**

To investigate clinical, immune, and metabolic parameters in response to tryptophan supplementation, in healthy subjects on a low tryptophan diet.

**Methods:**

We performed a randomized, double blind, placebo-controlled crossover study in 20 healthy volunteers (18 - 75 years old). Subjects were instructed to start a low tryptophan diet and then randomly assigned to a 3-week tryptophan supplementation (3g/day) or placebo, in enteric coated capsules. After a 2-week washout period, subjects crossed over to the opposite intervention arm. Questionnaires were used to assess bowel symptoms, anxiety, depression and stress levels (GSRS, HADS and DASS21, respectively). Stool, urine, blood and duodenal aspirates were collected to measure tryptophan metabolites and cytokines.

**Results:**

Tryptophan supplementation had no changes in gastrointestinal symptoms or behavioral parameters. Compared with placebo, tryptophan increased urinary and plasma levels of indoleamine 2,3-dioxygenase/kynurenine (p= 0.002 and p= 0.02, respectively) and indoles (p= 0.001 and p= 0.01, respectively), suggestive of activation of host and microbial metabolic pathways. Urinary and plasma metabolites were higher than in feces (p=<0.05), suggesting their active absorption in the small intestine. There were no differences in AhR activity in duodenal aspirates or in stool. Although no changes in the cytokine production were detected, serum kynurenine pathway metabolites negatively correlated with IL-8 levels (R=-0.72; p=0.001). Fecal tryptophan metabolites levels positively correlated with anxiety and depression scores, suggesting that the microbial metabolism of dietary tryptophan in the colon impacts host behavior.

**Conclusions:**

Tryptophan supplementation in healthy individuals was safe and had a measurable influence on microbial and host metabolism, mainly kynurenine and indole pathways, with known immunomodulatory properties. Tryptophan was metabolized and absorbed in the small intestine, reflected by the high metabolite levels in plasma and urine. Fecal metabolites correlating with clinical parameters reflect subjects’ long-term diet. Further studies are warranted to study tryptophan supplementation in disorders with altered AhR pathways.

**Funding Agencies:**

CIHR

